# Impaired glymphatic function and clearance of tau in an Alzheimer’s disease model

**DOI:** 10.1093/brain/awaa179

**Published:** 2020-07-23

**Authors:** Ian F Harrison, Ozama Ismail, Asif Machhada, Niall Colgan, Yolanda Ohene, Payam Nahavandi, Zeshan Ahmed, Alice Fisher, Soraya Meftah, Tracey K Murray, Ole P Ottersen, Erlend A Nagelhus, Michael J O’Neill, Jack A Wells, Mark F Lythgoe

**Affiliations:** a1 UCL Centre for Advanced Biomedical Imaging, Department of Imaging, Division of Medicine, University College London, London, UK; a2 Department of Neuroscience, Physiology and Pharmacology, University College London, London, UK; a3 School of Physics, National University of Ireland Galway, Ireland; a4 Eli Lilly and Company, Erl Wood Manor, Windlesham, Surrey, UK; a5 Office of the President, Karolinska Institutet, Stockholm, Sweden; a6 Institute of Basic Medical Sciences, University of Oslo, Oslo, Norway

**Keywords:** glymphatic, tau, aquaporin-4, Alzheimer’s disease, rTg4510

## Abstract

The glymphatic system, that is aquaporin 4 (AQP4) facilitated exchange of CSF with interstitial fluid (ISF), may provide a clearance pathway for protein species such as amyloid-β and tau, which accumulate in the brain in Alzheimer’s disease. Further, tau protein transference via the extracellular space, the compartment that is cleared by the glymphatic pathway, allows for its neuron-to-neuron propagation, and the regional progression of tauopathy in the disorder. The glymphatic system therefore represents an exciting new target for Alzheimer’s disease. Here we aim to understand the involvement of glymphatic CSF-ISF exchange in tau pathology. First, we demonstrate impaired CSF-ISF exchange and AQP4 polarization in a mouse model of tauopathy, suggesting that this clearance pathway may have the potential to exacerbate or even induce pathogenic accumulation of tau. Subsequently, we establish the central role of AQP4 in the glymphatic clearance of tau from the brain; showing marked impaired glymphatic CSF-ISF exchange and tau protein clearance using the novel AQP4 inhibitor, TGN-020. As such, we show that this system presents as a novel druggable target for the treatment of Alzheimer’s disease, and possibly other neurodegenerative diseases alike.

## Teaser

Harrison *et al.* show that regional variation in glymphatic function dictates tau accumulation in a mouse model of Alzheimer’s disease tauopathy. Modifying glymphatic flow by pharmacologically targeting aquaporin-4 alters tau clearance, suggesting therapeutic potential against tauopathies.

## Introduction

Impaired brain clearance mechanisms that result in the accumulation of aberrant proteins that define Alzheimer’s disease, provide new diagnostic and therapeutic opportunity to delay or prevent clinical symptoms. One such pathway for parenchymal protein clearance, the glymphatic system, has recently been described (Jessen *et al.*, 2015). Its involvement in Alzheimer’s disease, however, is yet to be fully defined. Alzheimer’s disease is a devastating condition, neuropathologically characterized by the extracellular accumulation of amyloid-β in the form of plaques, and intracellular accumulation of hyperphosphorylated microtubule associated protein tau in the form of neurofibrillary tangles (NFTs). Blood–brain barrier clearance, intra- and extracellular degradation, interstitial fluid (ISF) bulk flow clearance and CSF absorption pathways have all been implicated in removal of parenchymal amyloid-β (Zlokovic *et al.*, 2000; Glabe, 2001; Weller *et al.*, 2008; Iliff *et al.*, 2012; Hawkes *et al.*, 2014), while tau is thought to be predominantly cleared by degradation, ISF bulk flow, and CSF absorption clearance mechanisms (Chesser *et al.*, 2013; Iliff *et al.*, 2014). Recent evidence, however, has suggested that the glymphatic system may contribute to a larger portion of parenchymal clearance of these protein species than previously thought (Iliff *et al.*, 2012; Kress *et al.*, 2014; Tarasoff-Conway *et al.*, 2015).

The glymphatic clearance pathway was so named for its appropriation of the lymphatic function of interstitial protein management, and its dependence upon glial water transport (Jessen *et al.*, 2015). Hence, it describes the exchange of para-arterial CSF with ISF in the parenchymal extracellular space, and para-venous clearance out of the brain (Iliff *et al.*, 2012; Xie *et al.*, 2013; Holter *et al.*, 2017). Exchange of CSF with ISF is thought to be facilitated by the expression of aquaporin 4 (AQP4) channels on astrocytic endfeet; animals lacking AQP4 exhibit a ∼70% reduction in CSF influx and a ∼55% reduction in parenchymal solute clearance (Iliff *et al.*, 2012; Mestre *et al.*, 2018). Furthermore, deletion of AQP4 in APP/PS1 mice has been shown to potentiate the development of amyloid-β pathology and memory deficits (Xu *et al.*, 2015), suggesting that supressed glymphatic clearance is capable of inducing and/or advancing Alzheimer’s disease pathology. It has also been shown that the appropriate expression pattern of AQP4 polarization to astrocytic endfeet is required for efficient glymphatic CSF-ISF exchange (Iliff *et al.*, 2014; Kress *et al.*, 2014; Hadjihambi *et al.*, 2019), and that this polarization in the brain declines with age (Kress *et al.*, 2014; Zeppenfeld *et al.*, 2016), the greatest risk factor for developing Alzheimer’s disease (Guerreiro and Bras, 2015). Moreover, it was shown recently that AQP4 polarization itself is associated with Alzheimer’s disease status (Zeppenfeld *et al.*, 2016), suggesting that impaired glymphatic clearance may play a role in rendering the ageing brain vulnerable to aberrant protein deposition. Previous studies of glymphatic clearance in mouse models of Alzheimer’s disease however have focused largely on amyloid-β pathology (Peng *et al.*, 2016). In human Alzheimer’s disease though, it is the tau burden, not amyloid-β load, that predicts both brain atrophy as well as cognitive status in patients with Alzheimer’s disease (Giannakopoulos *et al.*, 2003). Furthermore, emerging evidence suggests that the extracellular space, which is cleared by the glymphatic system, acts as a conduit for neuron-to-neuron propagation and regional progression of Alzheimer’s disease tau pathology (Chesser *et al.*, 2013; Ahmed *et al.*, 2014; Medina and Avila, 2014; Lewis and Dickson, 2016; Wu *et al.*, 2016). This raises the intriguing possibility that reduced glymphatic clearance of tau may potentiate disease progression via exacerbated neuron-to-neuron propagation of tangle susceptible tau protein, and as such, would be a powerful target for therapy.

Here we study the glymphatic system using MRI, the clearance of parenchymal tau using intracerebral injections and CSF sampling, and the modulation of AQP4 function, in a mouse model that develops tau NFT pathology (Ramsden *et al.*, 2005) similar to that seen in neurodegenerative diseases such as Alzheimer’s disease (Braak and Braak, 1995). By using contrast-enhanced MRI, we provide a spatial and temporal description of the glymphatic system in the mouse brain, highlighting the heterogeneous nature of glymphatic inflow in the cortex, alluding to the critical role of this clearance system in the deposition of tau protein in the brain. We assess the function of glymphatic clearance in rTg4510 mice and their ability to clear parenchymal tau protein, as well as the expression pattern of AQP4 in affected regions. Finally, we demonstrate the critical role of this water channel, by pharmacologically inhibiting its function, and assessing the impact of such an inhibition on CSF-ISF exchange and tau clearance. In this study we provide the first demonstration of impaired glymphatic function in an animal model of tauopathy and establish the essential role of AQP4 in the clearance of tau protein from the brain.

## Materials and methods

### Animals and drugs

Generation of homozygous rTg4510 transgenic mice has been described previously (Ramsden *et al.*, 2005). The rTg4510 and litter matched wild-type mice were licensed from the Mayo Clinic (Jacksonville, Florida, USA), bred for Eli Lilly by Taconic, and imported into the UK for study at UCL’s Centre for Advanced Biomedical Imaging. For TGN-020 experiments, C57BL/6 mice were used, imported from Charles River. For additional TGN-020 experiments, where stated, AQP4 knockout mice, generation of which has been described previously (Thrane *et al.*, 2011) (henceforth referred to as *Aqp4*^−/−^ mice) were used, imported directly from University of Oslo, Norway, into the UK for study at UCL’s Centre for Advanced Biomedical Imaging. In all other experiments, unless stated otherwise, female mice of 8.5 months of age were used. Animals were housed in groups of three to five in individually ventilated cages with *ad libitum* access to standard mouse chow pellets, drinking water, and environmental enrichment, on a 12-h light/dark cycle. All animal work was performed in accordance with the UK’s Animals (Scientific Procedures) Act of 1986 and was previously approved by UCL’s internal Animal Welfare and Ethical Review Body.

For pharmacological inhibition of AQP4, TGN-020 (*N*-1,3,4-thiadiazol-2-yl-3-pyridinecarboxamide) (Tocris Bioscience) was used (Huber *et al.*, 2009). To increase solubility, TGN-020 was dissolved in a cyclodextrin derivative, 20% w/v Captisol^®^ (CyDex Pharmaceuticals) in water for injection. For TGN-020 experiments, mice were treated intraperitoneally with either TGN-020 (250 mg/kg in 20 ml/kg body weight) or vehicle (empty 20% Captisol^®^, 20 ml/kg).

### Tau immunohistochemistry

Immunohistochemistry was performed in order to quantify cortical deposition of tau in young rTg4510 mice. Female rTg4510 mice from 3.2 to 4.7 months of age were examined. Mice were perfused with PBS before removing and hemisecting the brain. The right hemisphere was drop fixed in 10% buffered formalin before being processed using the Tissue TEK VIP processor (GMI Inc) and embedded in paraffin wax. Sections (6 μm) of the brain in the sagittal plane were collected using a rotary microtome and mounted on glass slides. Following deparaffinization and rehydration of the tissue sections, antigen retrieval was carried out using the Lab Vision PT module system (Thermo Scientific), where sections were heated to 100°C for 20 min in citrate buffer (TA-250-PM1X; Thermo Scientific). Slides were transferred to a Lab Vision Autostainer (Thermo Scientific) where the following incubations were performed: 10 min in H_2_O_2_ (0.3%); 30 min in normal goat serum (1:20; Vector Laboratories); 60 min in primary antibody for tau phosphorylated at serine 409 (PG-5; 1:8000 from Peter Davies, Albert Einstein College of Medicine, NY, USA); 30 min in biotinylated goat anti-mouse IgG (1:200, BA9200; Vector Laboratories); 30 min avidin-biotin complex solution (PK-7100; Vector Laboratories); and 5 min in 3,3′-diaminobenzidine (SK-4105; Vector Laboratories). Apart from the last two steps, PBS with 0.05% Tween-20 (PBS-T) was used for diluting reagents and washes between steps. Sections were then counterstained with haematoxylin before dehydration and coverslipping. To quantify PG-5-positive tau pathology, stained sections were digitized using the Scanscope AT slide scanner (Aperio) at 20× magnification. Imagescope software (version 11.1.2.780; Aperio) was used to view the digitized tissue sections and delineate boundaries of the rostral and caudal cortex, and somatomotor and visual cortex areas. PG-5 immunoreactivity was quantified using the positive pixel algorithm (Imagescope, version 11.1.2.780; Aperio), and performed in a blinded fashion. Data are expressed as the ratio of percentage coverage of the total delineated boundary for rostral: caudal cortex, and somatomotor: visual cortex.

### Dynamic contrast-enhanced MRI

#### Surgical preparation

Mice were anaesthetized with 2% isoflurane delivered in O_2_ at a delivery rate of 1 l/min, and positioned in a stereotaxic frame with the head flexed to 50°. A midline incision was made at a midpoint between the skull base and the occipital margin to the first vertebrae. The underlying muscles were parted to expose the atlanto-occipital membrane and dura mater overlaying the cisterna magna, and a durotomy was performed using a 23-gauge needle. An intrathecal catheter (35–40 mm port length, 80–90 mm intravascular tippet length, Sandown Scientific) extended with polyethylene tubing (0.4 mm × 0.8 mm, Portex) and attached to a 50 µl glass Hamilton syringe driven by a microinfusion pump (sp210iw syringe pump, World Precision Instruments) was filled with low molecular weight paramagnetic contrast agent Magnevist^®^ (21 mM Gd-DTPA, molecular weight 938 Da; Schering Health Care Ltd, in filtered 0.9% NaCl). The catheter was advanced 1 mm into the cisternal space, sealed and anchored in place using superglue and fast setting resin (Araldite^®^).

#### MRI acquisition

Following surgery, animals were transferred to an MRI-compatible cradle with head held prone and a snout mask positioned to deliver 1.5% isoflurane in O_2_ at a delivery rate of 1 l/min. Core temperature and respiratory rate were monitored using a rectal probe and pressure pad, respectively (SA Instruments). Mice were maintained at 37°C (±2°C) using heated water tubing and a feedback loop controlled warm air blower (SA Instruments). Respiration rate was maintained between 80 and 120 breaths per minute by incrementally adjusting isoflurane dose. All imaging was performed with a 9.4 T VNMRS horizontal bore MRI scanner (Agilent Inc). A 72-mm inner diameter volume coil (Rapid Biomedical) was used for RF transmission and signal was received using a two-channel array head coil (Rapid Biomedical). A 3D T_1_-weighted gradient echo sequence was used to detect the motion of the Gd-DTPA with parameters: repetition time = 15 ms, echo time = 3.4 ms, flip angle = 15°, number of acquisitions = 3, field of view = 1.28 × 1.28 × 1.92 cm, scanning time = 12 min, acquisition matrix size of 128 × 128 × 128, yielding an image resolution of 0.1 × 0.1 × 0.15 mm. Three baseline scans were acquired prior to intrathecal infusion of Gd-DTPA via the indwelling catheter (30 μl at 0.6 μl/min, total time 50 min). Magnetic resonance images were continually acquired throughout and after intrathecal infusion for a total time of 180 min. At the end of the experiment the animal was euthanized by overdose with sodium pentobarbital (10 ml/kg, i.p.).

#### Image processing and analysis

First, the acquired T_1_-weighted MRI images were converted to the 3D NIfTI image format. Second, scan-to-scan misregistration caused by head movement was corrected by rigid-body alignment of each scan to the baseline averaged volume. Third, image intensity was normalized over the time series by estimating the intensity non-uniformity in the baseline volume and correcting all the volumes in the time series using the baseline intensity non-uniformity map, followed by 0.1 mm full-width at half-maximum isotropic Gaussian voxel-wise image intensity smoothing. For presentation of images, a difference image was calculated for each time point using the following expression:

(1) D(i,j,k) = [I(i,j,k) – B(i,j,k)]

where *D* is the difference image, *I* is the time series image post Gd-DTPA infusion, *B* is the average baseline image, and (*i, j, k*) represents the voxel position. Signal intensity measured on the T_1_-weighted magnetic resonance images over time in preselected anatomical areas were used to obtain intensity measurements. Both the T_1_-weighted averaged baseline images and the contrast-enhanced T_1_-weighted magnetic resonance images were used to anatomically guide placement of 3D regions of interests. The intensity signal for each region of interest on each time-point image was extracted and expressed as a % change from the average baseline image. For calculation of kinetic parameters such as the maximal intensity and the time at which half maximal intensity was achieved in each brain region, group signal intensity versus time data were fitted to a sigmoidal model:

(2) $$y=\frac{{\mathrm{Intensity}}_{\mathrm{Max}}}{1+e^{\left(\frac{{\mathrm{Time}}_{50}-\;x}{\mathrm{Slope}}\right)}}$$

where *Intensity_Max_* is the maximal intensity achieved in the brain region, *Time_50_* is the time at which half maximal intensity is achieved, and *Slope* is the gradient of the linear portion of the fitted sigmoidal curve. Additionally, each region of interest *Intensity_Max_* value was divided by its corresponding *Time_50_* value to give a measure of ‘Penetration Efficiency’ for each brain region. The value of each of these parameters was extracted from the best-fit curve of each group dataset, and are presented with their associated 95% confidence interval (CI).

### Intracerebral infusion of tau and quantification of parenchyma-CSF tau clearance

#### Preparation and characterization of tau-containing brain homogenate

Tau for intracortical injection was prepared by euthanizing an aged rTg4510 mouse (12 months of age) by overdose with sodium pentobarbital (10 ml/kg, i.p.) and dissecting out the cortex and hippocampus. Tissue was quickly frozen in isopentane on dry ice for storage at −80°C. Frozen tissue was weighed, thawed, and gently mixed in a mortar with a few strokes of a pestle in 10% w/v volumes of cold Tris-buffered saline (TBS) containing protease inhibitor cocktail, phosphatase inhibitor cocktails I and II (Sigma), at a final dilution of 1:100, and 1 mM phenylmethylsulphonyl fluoride. The resultant homogenate was centrifuged at 3000 rpm at 4°C for 5 min, and the pellet discarded. The supernatant was stored at −80°C until characterization and subsequent intracerebral infusion. Total and phosphorylated tau content was quantified by using ELISAs [Human Tau (total) (#KHB0041, Invitrogen) and Human Tau (Phospho) [pS_199_] ELISA Kits, as per the manufacturer’s instructions]. The ratio of phosphorylated (pS_199_) to total tau in the homogenate was 0.32 (±0.03). The ratio of insoluble to soluble tau in the homogenate was assessed by separating proteins by centrifugation (13 400 rpm at 4°C for 20 min) and quantifying tau [ELISA, Human Tau (total), as above] in the pellet (insoluble) and supernatant (soluble) fractions. The ratio of insoluble to soluble total tau in the homogenate was 0.14 (±0.01). Prior to intracerebral infusion, homogenate (total) tau concentration was adjusted to 20 μg/ml with homogenization buffer.

#### Intracerebral infusion

Mice were anaesthetized with 2% isoflurane delivered in O_2_ at a delivery rate of 1 l/min, and positioned in a stereotaxic frame in the horizontal skull position. A midline incision was made on the top of the head to expose the underlying skull. A small burr hole was made, using a microdrill, above the location of the intracerebral injection. Tau (50 ng) containing brain homogenate (see above for preparation) was infused into either the rostral (anteroposterior, +2 mm, and mediolateral, +2 mm relative to bregma, and ventrodorsal, −0.75 mm, relative to the brain surface) (Paxinos and Franklin, 2013) or caudal (anteroposterior, −2 mm, and mediolateral, +2 mm relative to bregma, and ventrodorsal, −0.75 mm, relative to the brain surface) (Paxinos and Franklin, 2013) cortex, or the striatum (anteroposterior, −0.2 mm, and mediolateral, +2 mm relative to bregma, and ventrodorsal, −1.75 mm, relative to the brain surface) (Paxinos and Franklin, 2013) using a 10 μl glass Hamilton syringe (2.5 μl at 0.5 μl/min, total time 5 min). Either 15, 30, or 60 min after the start of the intracerebral infusion, with the needle left *in situ*, a midline incision was made at a midpoint between the skull base and the occipital margin to the first vertebrae. The underlying muscles were parted to expose the atlanto-occipital membrane and dura mater overlaying the cisterna magna, which was thoroughly cleaned with saline. A durotomy was performed using a 23-gauge needle, allowing CSF to be collected using a narrow bore pipette tip. Volume of CSF collected varied between genotype: 5–8 µl was routinely collected from wild-type mice, whereas 10–15 µl was routinely collected from rTg4510 mice. At the end of the experiment the animal was euthanized by overdose with sodium pentobarbital (10 ml/kg, i.p.).

#### Confirmation of lack of blood contamination of CSF samples

The collected CSF was centrifuged briefly to pellet any red blood cell contaminants. The supernatant was removed and frozen at −20°C until further analysis by ELISA. Water (2 µl) was added to the blood pellet and snap-frozen on dry ice to ensure hypotonic freeze-thaw release of haemoglobin from any red blood cells present. The same sample preparation was performed on a volume (approximately equal to the volume of CSF extracted from each animal) of whole blood. Each thawed sample was measured at 417 nm on a NanoDrop ND-1000 spectrophotometer (Thermo Fischer Scientific) to quantify percentage contamination of each CSF sample. This method allows for measurement of blood contamination down to 0.0001%, well below that detectable by eye (∼0.01%). Lack of significant blood contamination was verified by an average percentage contamination of 0.003% (±0.0006%).

#### Tau ELISAs

Concentration of human tau in CSF samples was quantified using ELISAs. CSF total tau was quantified using Human Tau (total) ELISA Kit (#KHB0041, Invitrogen), and CSF phosphorylated tau was quantified using Human Tau (Phospho) (pS_199_) ELISA Kit (#KHB7041, Invitrogen), as per the manufacturer’s instructions. Briefly, CSF samples were diluted in diluent buffer prior to being incubated in capture antibody-coated wells for 2 h at room temperature. Wells were washed several times before being incubated in detection antibody for 1 h at room temperature. Wells were washed again before being incubated with horseradish peroxidase conjugated secondary antibody for 30 min at room temperature. Wells were then washed again before being incubated with stabilized chromogen for 30 min at room temperature. After this incubation, stop solution was added to each well and the plate was read at 450 nm. A set of standards of known (p)Tau concentration [0, 31.25, 62.5, 125, 250, 500, 1000, 2000 pg/ml for total tau, and 0, 15.625, 31.25, 62.5, 125, 250, 500, 1000 pg/ml for phospho (pS_199_) tau], were run in parallel for each experiment for quantification of CSF sample tau content from the standard curve.

### GFAP and AQP4 expression

#### Messenger RNA expression

For quantification of *Gfap* and *Aqp4* mRNA expression in regions of interest, mice were euthanized by overdose with sodium pentobarbital (10 ml/kg, i.p.), the brain rapidly removed and snap-frozen in isopentane pre-chilled on dry ice. Sagittal sections (25 μm) of the brain were collected onto RNase-free SuperFrost^®^ Plus slides, using a cryostat. Brain tissue from regions of interest or total whole brain tissue were isolated from five sagittal sections [∼1.0, 1.5, 2.0, 2.5 and 3.0 mm lateral to the midline (Paxinos and Franklin, 2013)] using a PALM MicroBeam laser capture microdissection system (Zeiss). Samples for each brain region for each animal were pooled and total RNA extracted using the RNeasy^®^ Plus Microkit (Qiagen) as per the manufacturer’s instructions. Total RNA was converted to cDNA using the QuantiTect^®^ Reverse Transcription Kit (Qiagen), which was then quantified using Eppendorf Mastercycler with Realplex software (v1.5, Eppendorf) and the TaqMan^®^ Gene Expression master mix (Applied Biosystems). TaqMan^®^ Gene Expression assays for each gene of interest (*Gfap* and *Aqp4*) and reference genes [*Actb* (β-actin) and *Gapdh*) were used, and gene expression of genes of interest determined using the 2^−ΔΔCt^ method normalizing to expression in the wild-type whole brain.

#### Protein expression

For quantification of GFAP and AQP4 protein expression in regions of interest, mice were perfused with PBS before removing and hemisecting the brain. The right hemisphere was drop fixed in 10% buffered formalin before being processed using the Tissue TEK VIP processor (GMI Inc.) and embedded in paraffin wax. Sections (6-μm thick) of the brain in the sagittal plane were collected using a rotary microtome and mounted on glass slides. The following primary antibodies were used for immunohistochemistry: rabbit monoclonal GFAP (1:3000; PU020-UP, Biogenix) and mouse monoclonal AQP4 (1:200; ab9512, Abcam). In brief, tissue sections were deparaffinized and rehydrated before antigen retrieval was performed using the Labvision PT module system (Thermo Scientific), heating slides to 100°C in citrate buffer (TA-250-PMX1, Thermo Scientific) for 20 min. Slides were then cooled in dH_2_O and transferred to a Lab Vision autostainer (Thermo Scientific) where the following steps were performed: 10 min in 0.3% H_2_O_2_; 30 min in normal goat serum (1:20; S-1000, Vector Labs); 60 min in primary antibody; 30 min in biotinylated goat anti-rabbit (1:200; BA-1000, Vector Labs) or goat anti-mouse (1:200; BA-9200, Vector Labs) secondary antibodies; 30 min in avidin-biotin complex solution (PK-7100, Vector Labs); 5 min in 3,3′-diaminobenzidine (SK-4105, Vector Labs). Apart from the last two steps, PBS with 0.05% Tween-20 (PBS-T) was used for diluting reagents and washes between steps. Sections were then counterstained with haematoxylin before dehydration and coverslipping. Stained sections were digitized using the Scanscope AT slide scanner (Aperio) at 20× magnification. ImageJ software (version 1.44p) was used to view and uniformly threshold for immunoreactivity. Regions of interest were drawn on each image for extraction of percentage immunopositive coverage (% immunoreactivity of whole brain region) for each region, in a blinded fashion.

### Cellular localization of AQP4

#### Immunofluorescence

Tissue sections for subcellular localization of AQP4 in relation to cerebral blood vessels or astrocytes were processed as above and immunofluorescently stained for AQP4 (1:60, ab9512, Abcam), and either the blood vessel endothelial cell marker CD31 (1:25, ab28364, Abcam), or astrocyte marker GFAP (1:3000; PU020-UP, Biogenix). Following deparaffinization and antigen retrieval, tissue sections were incubated in a cocktail of primary antibodies overnight at 4°C [CD31, 1:25 (ab28364, Abcam); GFAP (1:3000; PU020-UP, Biogenix); AQP4, 1:60 (ab9512, Abcam)], washed in PBS-T and then incubated in a cocktail of fluorophore-conjugated goat anti-rabbit (Alexa Fluor^®^ 488, 1:500; A11008) and goat anti-mouse (Alexa Fluor^®^ 568, 1:500; A11004) secondary antibodies (Invitrogen) for 2 h at room temperature. Finally, sections were washed in PSB-T and then dH_2_O, before being cover-slipped in VECTASHIELD^®^ mounting medium with DAPI (H-1200, Vector Labs). All incubations were performed manually and in a humidity tray, with minimal exposure to light. Immunofluorescence images were taken using a Leica DMLB fluorescent microscope and Q Capture Pro7 software (QImaging). Images were acquired by single excitation of each wavelength (separately) and channels subsequently merged.

#### Quantification of AQP4 expression across blood vessel cross-sections

Evaluation of the localization of AQP4 to perivascular endfeet and glial limitans was carried out by measuring the pixel intensities of CD31 and AQP4 immunoreactivity across cross-sections of blood vessels in each of the brain regions studied. For this, vessel containing regions of interest were identified on DAPI images: identified using flattened nuclei, clusters or lines of nuclei out of focus compared to the surrounding tissue and ‘negative’ space between nuclei without background fluorescence. Fluorescent intensity for both CD31 and AQP4 markers were measured across a single 4 µm axis perpendicular to the vessel orientation, and expressed as intensity, normalized to the intensity of a randomly selected background region of interest in the same image for each antigen, to generate linear plots of fluorescence extending from the brain tissue, into the vessel and again into the surrounding brain tissue.

#### Quantification of AQP4 polarization

Perivascular polarization of AQP4 was measured as previously described (Kress *et al.*, 2014). Briefly, the median immunofluorescence intensity of perivascular regions was measured. A threshold analysis was then used to measure the percentage of the region exhibiting AQP4 immunofluorescence greater than or equal to perivascular AQP4 immunofluorescence (AQP4% area). Polarization was expressed as the percentage of the region that exhibited lower AQP4 immunoreactivity than the perivascular endfeet. AQP4 vessel coverage was measured by firstly delineating the area of the vessel from the CD31 channel image. This region of interest was then placed on the AQP4 channel image thresholded for immunoreactivity for extraction of the percentage vessel coverage (% immunoreactivity of AQP4 of whole delineated vessel).

### Statistical analysis

Parameters extracted from sigmoidal fitting of MRI datasets are presented as best-fit values, with their associated 95% CI. All other data are represented as mean ± standard error of the mean (SEM) for the *n* number of animals in each group. Statistical comparisons between groups were performed via either a repeated measures two-way ANOVA followed by *post hoc* Bonferroni post-tests for multiple comparisons (for MRI data and immunofluorescence line profile data), or a regular two-way ANOVA followed by *post hoc* Bonferroni post-tests for multiple comparisons (for all other grouped comparisons not containing repeated measures). A one-way ANOVA was used for regional comparisons, and unpaired *t*-tests used for single comparisons. All statistical testing was performed using GraphPad Prism (v5 for Windows, San Diego, CA, USA).

### Data availability

Raw data and images are available upon request from the corresponding author.

## Results

### Heterogeneous deposition of tau in the cortex of the rTg4510 mouse

**Figure 1:**
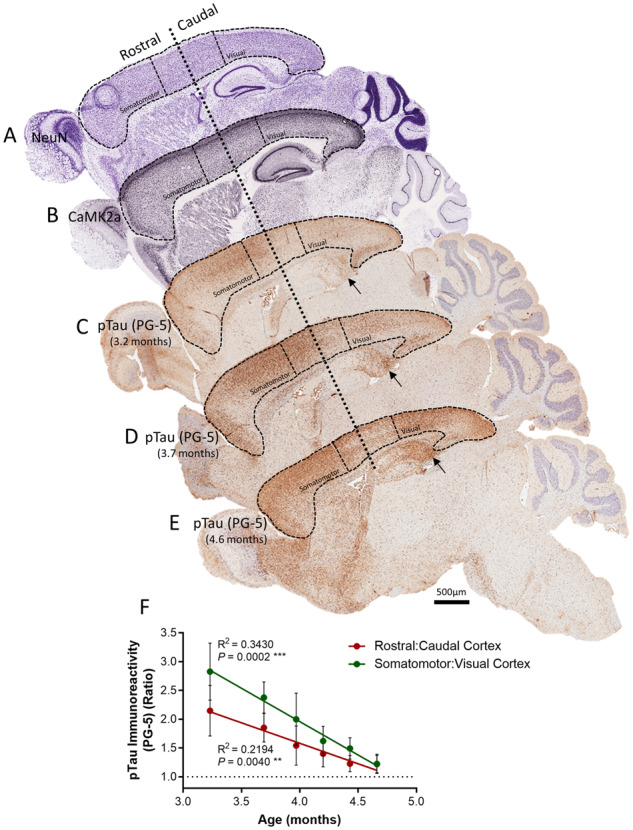
**Heterogeneous deposition of tau in the cortex of rTg4510 mice.** (**A**) NeuN and (**B**) CaMK2a expression in the mouse brain appear homogenous throughout rostral to caudal aspects of the cortex. During early deposition of tau in the rTg4510 mouse brain, however, pTau (PG-5) staining appears greater in the rostral compared to the caudal cortex, at (**C**) 3.2 months and (**D**) 3.7 months, subsiding by (**E**) 4.6 months of age. Subiculum of the hippocampus indicated with arrows on PG-5 staining images. Scale bar = 500 µm. (**F**) Quantification of the ratio of PG-5 immunoreactivity in the rostral and caudal cortex, and the somatomotor and visual cortex, reveal this pattern of elevated rostral compared to caudal tau deposition in the rTg4510 cortex during the early stages of deposition. *n *=* *5–7 per group.

Transgene expression in the rTg4510 mouse model is driven by the CaMK2a promoter and hence is restricted to the forebrain during the early stages of tau deposition. CaMK2a expression is notably homogenous throughout the cortex in the mouse brain (Fig. 1B) and hence the deposition of tau protein is hypothesized to be the same. PG-5 (Tau pS_409_) immunohistochemical analysis of 3.2 to 4.7-month-old rTg4510 mice, however, revealed greater rostral compared to caudal cortical tau pathology during early tau deposition in this model [rostral cortex PG-% immunoreactivity 5.98% (±0.67%) versus 3.03% (±0.49%) in the caudal cortex, in 3.2-month-old rTg4510 mice, rostral: caudal cortex PG-% immunoreactivity ratio, 2.15 (±0.44)] (Fig. 1C–F). Moreover when the ratio of rostral:caudal PG-5 immunoreactivity in extreme rostral/caudal aspects of the cortex is compared, i.e. the somatomotor versus visual cortex, the pattern of elevated rostral compared to caudal tau deposition becomes more apparent [somatomotor: visual cortex PG-% immunoreactivity ratio at 3.2 months, 2.83 (±0.50)] (Fig. 1F). This phenomenon, however, appears to subside with age, as mature NFT pathology takes hold [rostral:caudal cortex PG-% immunoreactivity ratio at 3.2 months, 2.15 (±0.44), compared to 1.22 (±0.15) at 4.7 months] (Fig. 1F). These data may indicate greater clearance of expressed phosphorylated tau in caudal compared to rostral aspects of the mouse cortex.

### Spatial and temporal profile of CSF-ISF exchange in the mouse brain

To investigate whether cortical heterogeneity of glymphatic CSF-ISF exchange could be related to the findings in Fig. 1, we analysed regional glymphatic inflow in the healthy wild-type mouse. By investigating the wild-type brain in this way, rather than the rTg4510 brain, we sought to investigate whether regional heterogeneity of CSF-ISF exchange in the healthy brain may predispose certain regions to aberrant protein accumulation. The function of the glymphatic pathway was investigated by quantifying the regional delivery pattern of a magnetic resonance contrast agent (Gd-DTPA, Magnevist^®^) into the brain from the CSF compartment, via contrast-enhanced MRI. As such Gd-DTPA was infused intrathecally (via the cisterna magna) and T_1_-weighted images serially acquired over 3 h (Supplementary Fig. 1A). This demonstrated a distinct pattern of CSF flow into the mouse brain, confirming the distribution of glymphatic flow previously observed in rodents (Supplementary Fig. 1B–D) (Iliff *et al.*, 2013*a*; Gaberel *et al.*, 2014). We observed a plateau in maximal MRI signal in all areas of the brain by ∼90 min post-initiation of intrathecal Gd-DTPA infusion (Supplementary Fig. 1D). Time_50_ values, the time at which half-maximal intensity was observed, indicated that the superficial brain regions examined exhibit a distinct chronological sequence of Gd-DTPA infiltration (Supplementary Fig. 1D and F). T_1_ contrast was initially observed in the CSF filled aqueduct, progressing to ventral nuclei (the pontine nucleus and pituitary recess) and the caudal cortex. Later time points showed advancement of the signal throughout the brain, in the pineal recess and cerebellum, and more rostral regions, such as the rostral cortex and the olfactory bulbs.

**Figure 2:**
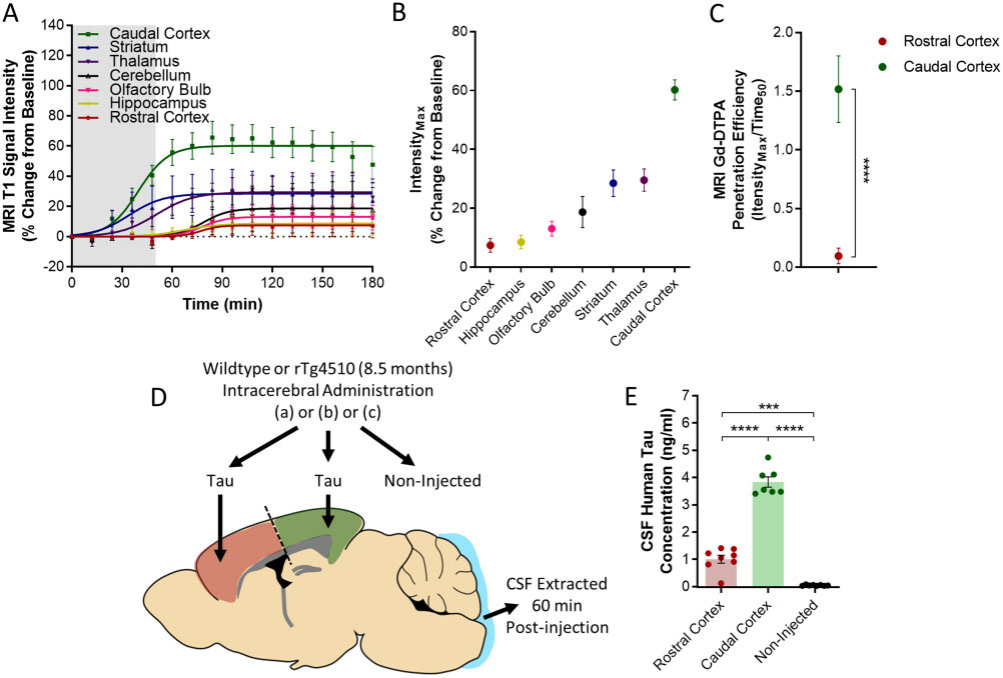
**Glymphatic inflow and clearance of tau from the mouse brain cortex.** (**A**) MRI T_1_ signal intensity versus time data acquired from grey matter regions of the mouse brain showing differences in rates and intensities of glymphatic inflow of Gd-DTPA in anatomically discreet regions over time. (**B**) Intensity_Max_ values (best-fit of maximal intensity achieved in region from sigmoidal fitting of data) in each of the brain regions in which raw data and sigmoidal curves are displayed in **A**. (**C**) MRI Gd-DTPA penetration efficiency (best-fit Intensity_Max_ divided by Time_50_ values) in the rostral and caudal cortex, demonstrating the heterogeneity of the extent of glymphatic inflow into these two regions of the mouse cortex. (**D**) Schematic illustrating brain homogenate injection experiments in which tau containing brain homogenate was injected into either the rostral or caudal cortex, and CSF collected from the cisterna magna 60 min later. (**E**) Tau concentration of CSF samples collected in experiments shown schematically in **D**, demonstrating greater clearance of tau from the caudal compared to rostral aspect of the mouse cortex. Mean ± SEM of data and fitted curves shown in **A**, raw data and mean ± SEM between animals shown in **E**, and best-fit value and associated 95% CI of sigmoidal fitting of data shown in **B** and **C**. *n *=* *5–8 per group. Statistical significance denoted by asterisks: ***P <* *0.001, ****P <* *0.0001.

Quantification of magnetic resonance signal in grey matter revealed heterogeneity of CSF-ISF exchange in the mouse brain (Fig. 2A–C). Consistent with the pattern of tau deposition in the cortex of tauopathy mice (Fig. 1), the rostral cortex of wild-type animals exhibited a relatively low level of Gd-DTPA infiltration [Intensity_Max_ (% change from baseline), 7.44% (95% CI, 5.76 to 9.11%)]. Yet the caudal cortex exhibited a high level of contrast agent infiltration [Intensity_Max_ (% change from baseline), 60.2% (95% CI, 57.8 to 62.6%)] (Fig. 2A and B). Moreover, MRI Gd-DTPA penetration efficiency (Intensity_Max_/Time_50_) demonstrated the heterogeneity of glymphatic CSF-ISF exchange in the cortex of wild-type animals even further [penetration efficiency in the caudal cortex, 1.51 (95% CI, 1.32 to 1.72), versus 0.10 (95% CI, 0.05 to 0.14) in the rostral cortex, *P *<* *0.0001] (Fig. 2C).

To investigate this heterogeneity further, tau containing brain homogenate was infused into either the rostral or caudal cortex prior to CSF tau sampling from the cisterna magna (Fig. 2D). We observed a similar pattern to that observed with MRI, in that more parenchymal tau was cleared into CSF from the caudal compared to the rostral cortex [CSF tau post infusion into the rostral and caudal cortex, 0.98 ng/ml (±0.18 ng/ml) and 3.80 ng/ml (±0.22 ng/ml) respectively, *P *<* *0.0001] (Fig. 2E).

Together these data suggest that glymphatic inflow in the wild-type mouse cortex, as quantified through contrast-enhanced MRI, corresponds well with the extent of parenchymal tau clearance, and that this is lower in the rostral compared to the caudal cortex, consistent with localization of early cortical tau deposition in transgenic tau animals.

### Altered CSF-ISF exchange in the rTg4510 mouse

**Figure 3:**
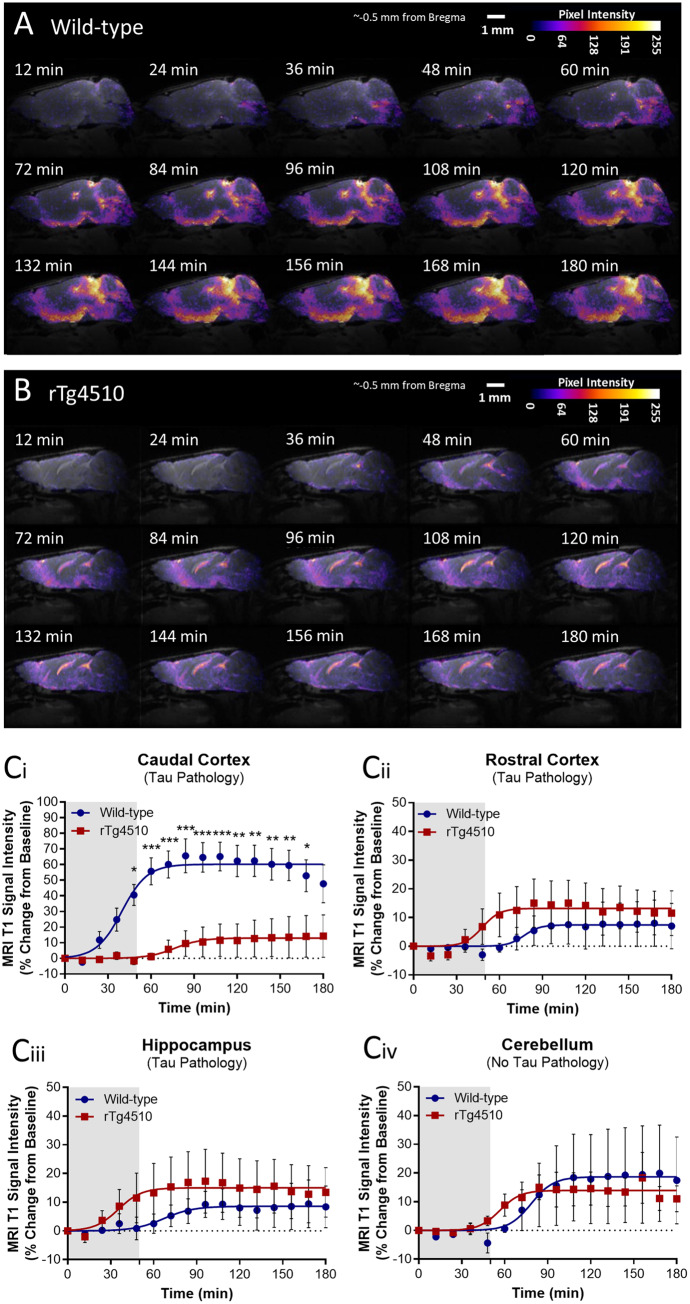
**Altered CSF-ISF exchange in rTg4510 mice.** (**A**) Representative pseudocolour scaled sagittal (∼0.5 mm lateral of bregma) images of a (**A**) wild-type and (**B**) rTg4510 mouse brain after cisterna magna infusion of Gd-DTPA, showing infiltration of contrast agent into the brain parenchyma. Scale bars = 1 mm. (**C**) MRI T_1_ signal intensity versus time data acquired from areas affected by tau pathology in the rTg4510 mouse model: the (**Ci**) caudal cortex, (**Cii**) rostral cortex and the (**Ciii**) hippocampus; and the (**Civ**) cerebellum, which is void of tau pathology in the mouse. Mean ± SEM of data and fitted curves shown. Extracted parameter of fitting displayed in Table 1. *n *=* *5 per group. Statistical significance denoted by asterisks: *P <* *0.05, **P <* *0.01, ***P <* *0.001.

To ascertain the function of the glymphatic clearance system in the scenario of mature tauopathy and neurodegeneration, cisternal delivery of Gd-DTPA and serial T_1_-weighted MRI acquisition was performed in rTg4510 animals (8.5 months of age) (Fig. 3A and B). At this age, significant mature NFT pathology is evident in the forebrain and is accompanied by neurodegeneration and cerebral atrophy (Ramsden *et al.*, 2005; Spires *et al.*, 2006; Holmes *et al.*, 2016). We observed notable impaired CSF-ISF exchange in the caudal cortex of transgenic tau mice, as indicated by a significant reduction of contrast agent penetration [Fig. 3C(i)], and reduced Intensity_Max_ and increased Time_50_ values (Table 1) in this region.

**Table 1:** Altered CSF-ISF exchange in rTg4510 mice

	Wild-type		rTg4510		Effect
	Best-fit value	95% CI	Best-fit value	95% CI	Significance
Caudal cortex					
Intensity_Max_ (%)	60.17	54.85–65.50	13.00	6.553–19.44	**↓ *P <* 0.0001** ****
Time_50_ (min)	39.66	32.38–46.94	76.50	41.31–111.7	**↑ *P <* 0.001** ***
Rostral cortex					
Intensity_Max_ (%)	7.432	3.671–11.19	13.18	9.163–17.20	**↑ *P <* 0.01** **
Time_50_ (min)	77.14	47.89–106.4	48.09	27.77–68.40	**↓ *P <* 0.01** **
Hippocampus					
Intensity_Max_ (%)	8.571	4.891–12.25	15.00	9.951–20.05	**↑ *P <* 0.01** **
Time_50_ (min)	67.04	33.83–100.2	35.35	8.222–62.48	**↓ *P <* 0.01** **
Cerebellum					
Intensity_Max_ (%)	18.67	10.37–26.97	13.94	11.08–16.79	↓ *P* > 0.05
Time_50_ (min)	78.49	49.56–107.4	56.63	42.29–70.98	↓ *P* > 0.05

Best-fit values and associated 95% CIs for Intensity_Max_ (the maximal intensity achieved in the brain region) and Time_50_ (the time at which half maximal intensity is achieved), extracted from sigmoidal fitting of wild-type and rTg4510 MRI T_1_ signal intensity versus time data presented in Fig. 3, showing opposing effects in the caudal cortex compared to the rostral cortex and hippocampus. *n *=* *5 per group. Statistical significance:

** *P < *0.01,

*** *P < *0.001,

**** *P < *0.0001.

In areas affected by tau pathology in the rTg4510 other than the caudal cortex, i.e. the rostral cortex and hippocampus (Ramsden *et al.*, 2005; Spires *et al.*, 2006; Holmes *et al.*, 2016), we did not observe any significant differences in signal intensities in time point by time point comparisons to wild-types [Fig. 3C(ii and iii)]. There were, however, subtle elevations in signal intensities in both of these regions in the rTg4510 mouse, which we investigated further. We observed increased Intensity_Max_ values [rTg4510 rostral cortex and hippocampus, 13.1 (95% CI, 9.16 to 17.2%) and 15.0 (95% CI, 9.95 to 20.0%), respectively versus 7.43 (95% CI, 3.67 to 11.2%) and 8.57 (95% CI, 4.89 to 12.3%) in wild-type animals, *P *<* *0.01 in both comparisons] (Table 1). Similarly, we observed reduced Time_50_ values [rTg4510 rostral cortex and hippocampus, 48.1 min (95% CI, 27.8 to 68.4 min) and 35.4 min (95% CI, 8.22 to 62.5 min), respectively, versus 77.1 min (95% CI, 47.9 to 106 min) and 67.0 min (95% CI, 33.8 to 100 min) in wild-type animals, *P *<* *0.001 in both comparisons] (Table 1), indicating subtle increases in CSF-ISF exchange in these tau burdened regions. It is worth noting that these regions present with markedly less CSF ingress in the wild-type mouse brain (∼7–8 fold lower), compared to other regions, which may account for the more subtle differences between animal groups observed.

In the cerebellum, which is not affected by tau pathology nor neurodegeneration (Ramsden *et al.*, 2005), no differences were observed in intensity versus time datasets [Fig. 3C(iv)], Time_50_ or Intensity_Max_ values (Table 1), between rTg4510 and wild-type animals, indicating lack of any change in CSF-ISF exchange in this region.

### Reduced CSF-ISF exchange, clearance of tau, and polarization of AQP4 in the rTg4510 mouse

**Figure 4:**
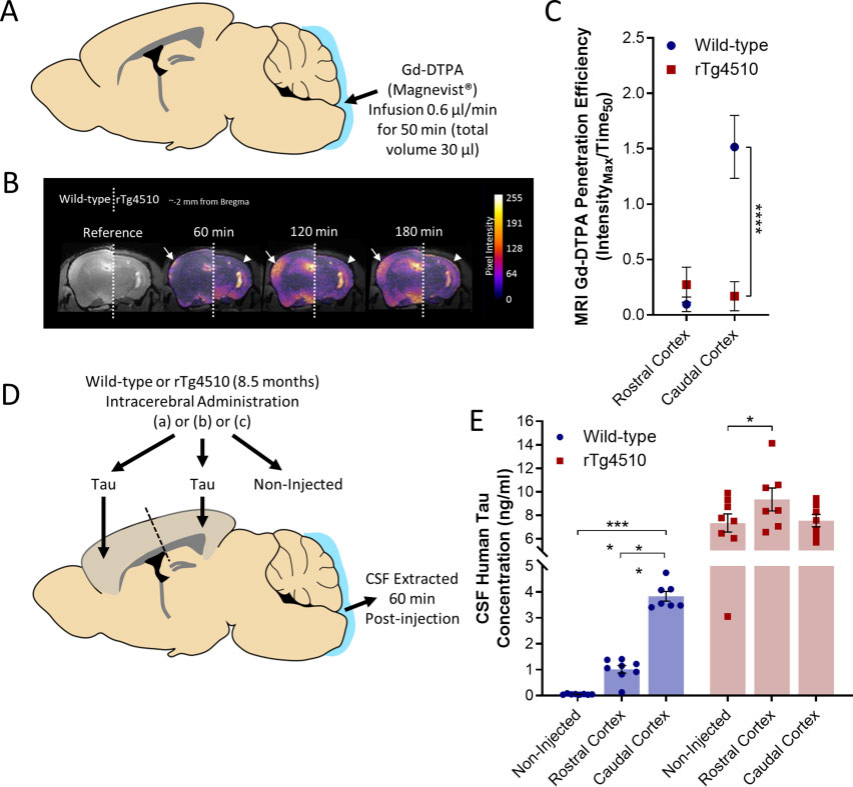
**Reduced glymphatic inflow and clearance of Tau in rTg4510 mice.** (**A**) Schematic illustrating infusion of Gd-DTPA into the cisterna magna of the mouse for quantification of glymphatic inflow in the brain. (**B**) Representative pseudocolour scaled coronal (∼−2 mm from bregma) images of the (*left*) wild-type and (*right*) rTg4510 mouse brain, highlighting the difference in extent of contrast agent infiltration into the caudal cortex [designated by white arrows (wild-type) and arrowheards (rTg4510)] over time. This difference is further exemplified through the calculated Gd-DTPA penetration efficiency data shown in **C**. (**D**) Schematic illustrating brain homogenate injection experiments in which tau-containing brain homogenate was injected into either the rostral or caudal cortex of wild-type and rTg4510 mice, and CSF extracted from the cisterna magna 60 min later. (**E**) Tau concentration of CSF samples extracted from experiments shown schematically in **D** demonstrating reduced clearance from the caudal cortex of rTg4510 mice compared to wild-type animals. Raw data and mean ± SEM between animals shown in **E**, and best-fit value and associated 95% CI of sigmoidal fitting of data shown in **C**. *n *=* *5–8 per group. Statistical significance denoted by asterisks: **P <* *0.01, ****P <* *0.0001.

Based on the observed differences in CSF-ISF exchange in the rTg4510 compared to wild-type mice, we focused further on the impairment observed in contrast-enhanced magnetic resonance data, as well as clearance of tau from the brain, and AQP4 polarization (Fig. 4). Penetration efficiency of the MRI contrast agent in both the caudal and rostral cortex (Fig. 4A) again demonstrated the impairment of glymphatic CSF-ISF exchange in the caudal cortex of the rTg4510 mouse [penetration efficiency in the caudal cortex of rTg4510 mice, 0.17 (±0.05), versus 1.52 (±0.10) in the wild-type animals, *P *<* *0.0001] (Fig. 4C). This reduction in contrast agent infiltration into the caudal cortex of rTg4510 mice was particularly evident in coronal MRI (Fig. 4B, arrow highlighting penetration in wild-type brains, arrowhead indicating lack of penetration in rTg4510 animals).

To assess whether or not such a reduction in CSF-ISF exchange also translated to a reduction in clearance of tau from the rTg4510 brain, tau containing brain homogenate was infused into either the rostral or caudal cortex of transgenic mice (Fig. 4D). CSF tau content revealed a similar pattern to contrast-enhanced magnetic resonance data. As previously noted, following infusion of tau into the cortex in wild-type animals, greater clearance was observed from the caudal as opposed to the rostral cortex (Figs 2E and 4E). Compared to non-injected controls, after rTg4510 mice receive infusion of tau into the rostral cortex, greater tau CSF concentration was observed indicative of tau parenchymal-CSF tau clearance [tau concentration in CSF following rostral cortex infusion in rTg4510 mice, 7.55ng/ml (±0.53 ng/ml) versus 7.35 ng/ml (±0.77 ng/ml) in non-injected rTg4510 mice, *P *<* *0.05]. However, after rTg4510 mice receive infusion of tau into the caudal cortex, no difference was observed in CSF tau concentration suggestive of compromised tau clearance (Fig. 4E). It should be noted that rTg4510 mice exhibit a far higher level of human tau in the circulating CSF, due to expression of the mutant tau transgene in the brain, and hence comparisons in these mice have been made to non-injected rTg4510 animal CSF rather than to wild-type mouse CSF data.

**Figure 5:**
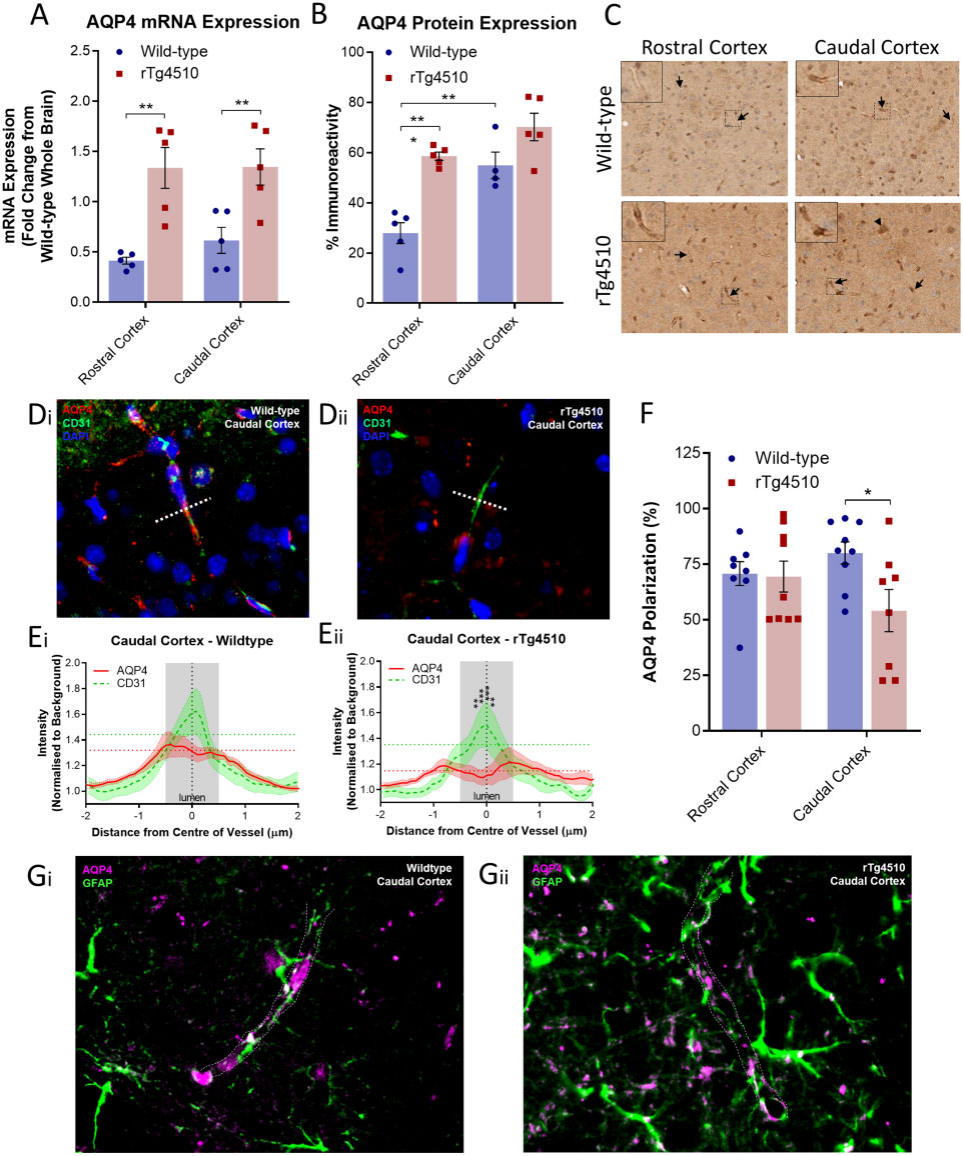
**AQP4 expression and polarization in rTg4510 mice.** Quantification of (**A**) mRNA and (**B**) protein expression of AQP4 in the rostral and caudal cortex of wild-type and rTg4510 mice, demonstrating upregulation in rTg4510 mice compared to wild-type controls. (**C**) Representative example images of brain tissue from wild-type and rTg4510 mice immunohistochemically stained for AQP4. Arrows indicate examples of immunopositive blood vessels in each image, which are shown at greater magnification in *insets*. (**D**) Representative immunofluorescence images of blood vessels in the caudal cortex of (**Di**) wild-type, and (**Dii**) rTg4510 mice stained for AQP4 and CD31, illustrating placement of 4-µm axis perpendicular to blood vessels for quantification of expression across vessel cross-sections, illustrating reduced AQP4 expression surrounding blood vessels in the caudal cortex of rTg4510 mice (**E**). (**F**) Quantification of AQP4 polarization similarly demonstrated reduced polarization to blood vessels in the caudal cortex of rTg4510 mice. This is further exemplified in caudal cortex sections stained for AQP4 and GFAP, demonstrating reduced endfoot AQP4 vessel coverage in the rTg4510 caudal cortex compared to wild-type (**G**). *n *=* *5–9 per group. Statistical significance denoted by asterisks: *P <* *0.05, **P <* *0.01, ***P <* *0.001.

Assessment of astrocytic expression and polarization of AQP4, an essential component of the glymphatic system, revealed differences in line with MRI contrast agent infiltration and tau clearance from the brain. *Aqp4* mRNA expression was observed to be upregulated in both the rostral and caudal cortex of the rTg4510 brain (Fig. 5A), consistent with the extent of astrogliosis observed as a result of cortical NFT pathology (Supplementary Fig. 2) (Ramsden *et al.*, 2005; Spires *et al.*, 2006; Holmes *et al.*, 2016). However, in the caudal cortex, this increased mRNA expression did not result in significant upregulated expression of AQP4 protein (Fig. 5B and C). On the contrary, in line with the MRI observation in wild-type animals of greater contrast agent infiltration in the caudal compared to rostral cortex, significantly greater AQP4 protein expression was observed in the caudal compared to the rostral cortex [AQP4 immunoreactivity in the caudal cortex 54.9% (±5.28%), versus 27.9% (±4.17%) in the rostral cortex, *P *<* *0.01]. Analysis of vessel cross-sections (Fig. 5D) from the caudal cortex of rTg4510 animals also revealed impaired AQP4 expression surrounding blood vessels (AQP4 expression lower than CD31 in the central 0.25 μm portion of blood vessels, *P *<* *0.01) [Fig. 5E(ii)]. This reduced perivascular localization of AQP4 in the rTg4510 caudal cortex was concordant with a reduction of ‘polarization’ of this water channel to blood vessels [AQP4 ‘polarization’ in the caudal cortex of wild-type mice, 80.0% (±4.96%) versus 54.1% (±9.48%) in the caudal cortex of rTg4510 animals] (Fig. 5F). This reduced polarization was further evident when caudal cortex tissue was double immunofluorescently stained for AQP4 and GFAP, demonstrating reduced endfoot AQP4 vessel coverage in the rTg4510 caudal cortex compared to wild-type (Fig. 5G).

Together, these data provide evidence that astrocytic AQP4 expression and its polarized localization are similarly perturbed in a region of the rTg4510 mouse brain, which exhibits impaired glymphatic inflow, and clearance of tau.

### Effect of pharmacological inhibition of AQP4 on CSF-ISF exchange and clearance of tau

**Figure 6:**
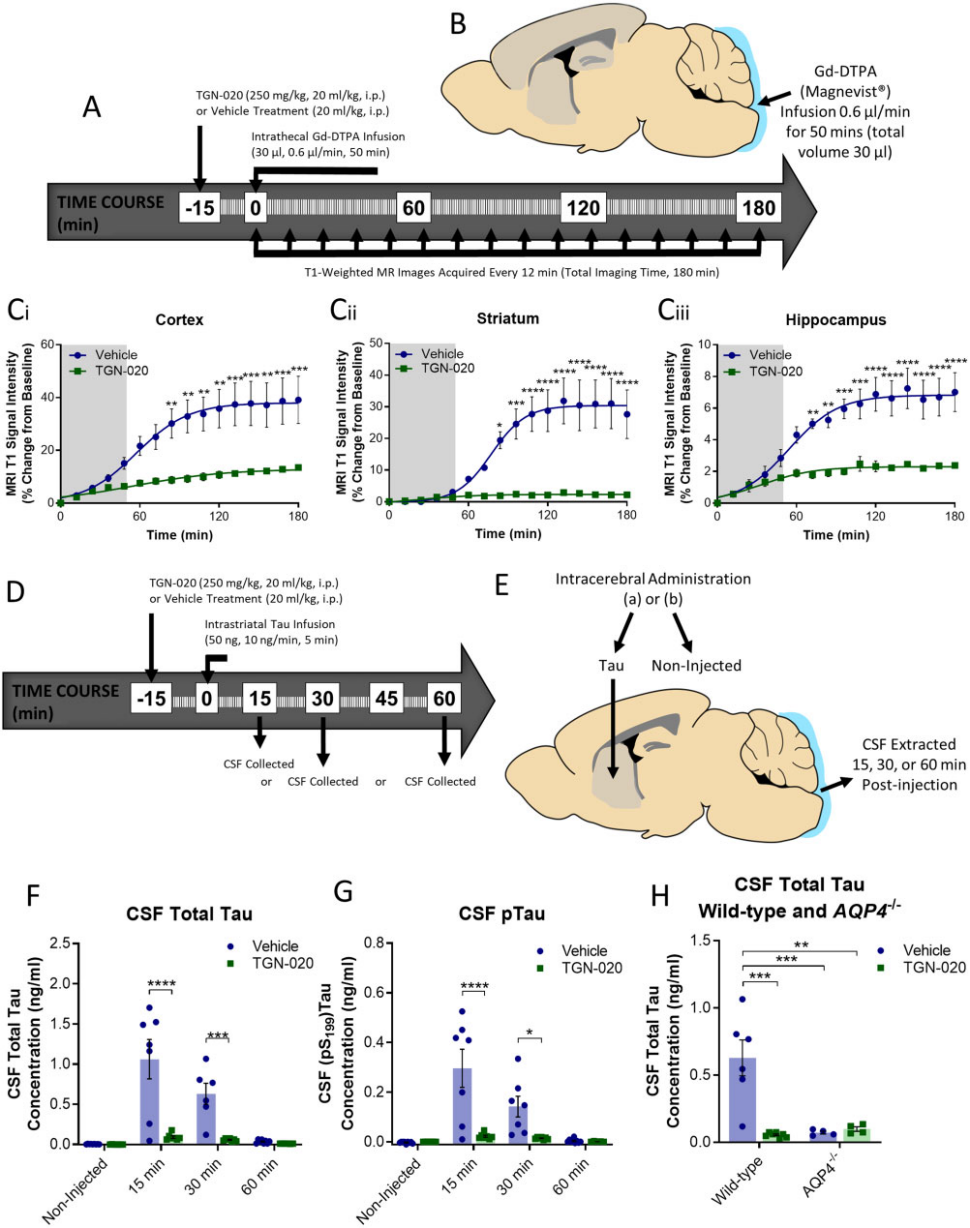
**Effect of pharmacological inhibition of AQP4 on CSF-ISF exchange and clearance of tau.** (**A**) Timeline and (**B**) schematic illustrating experiments used to determine the effects of pharmacological inhibition of AQP4 on glymphatic inflow in the mouse brain. (**C**) MRI T_1_ signal intensity versus time data acquired from the (**Ci**) cortex, (**Cii**) striatum, (**Ciii**) hippocampus during these experiments, demonstrating significant inhibition of glymphatic inflow. (**D**) Timeline and (**E**) schematic illustrating experiments used to determine the effects of pharmacological inhibition of AQP4 on clearance of tau from the mouse brain. (**F**) Total tau and (**G**) pTau (pS_199_) concentration of CSF samples extracted from experiments shown schematically in **D** and **E** demonstrating reduced clearance of tau from the TGN-020 treated animal brain. Experiments repeated in a cohort of *Aqp4*^−/−^ animals after TGN-020 or vehicle treatment, and CSF extracted 30 min post-injection for quantification of tau in CSF extracts. (**H**) CSF tau concentrations demonstrate the specific nature of TGN-020 towards AQP4, given the lack of an effect observed of TGN-020 in *Aqp4*^−/−^ animals. Mean ± SEM of data and fitted curves shown in **C**, raw data and mean ± SEM between animals shown in **F**, **G** and **H**. *n *=* *4–7 per group. Statistical significance denoted by asterisks: **P <* *0.01, ***P <* *0.001, ****P <* *0.0001.

Based on these altered AQP4 expression and polarization profiles, the role of AQP4 in glymphatic inflow and clearance of tau was further investigated, via its pharmacological blockade, with a specific inhibitor, TGN-020 (Huber *et al.*, 2009). In a cohort of wild-type mice, TGN-020 or vehicle were administered 15 min prior to cisternal infusion of Gd-DTPA, and serial acquisition of T_1_-weighted MRI for assessment of CSF-ISF exchange (Fig. 6A and B). Mice treated with TGN-020 exhibited significant reductions in magnetic resonance signal enhancement in the brain (Fig. 6C). This was clearly evident in the cortex [Fig. 6C(i)], striatum [Fig. 6C(ii)] and hippocampus [Fig. 6C(iii)], with significantly reduced magnetic resonance signal (*P *<* *0.05) being observed in TGN-020 treated animals from ∼80 min after the start of contrast agent infusion.

To determine if pharmacological blockade of AQP4 exhibited a similar reduction in clearance of tau from the brain, wild-type mice were treated with either TGN-020 or vehicle, 15 min prior to intrastriatal infusion of tau containing brain homogenate (Fig. 6D and E). The striatum was chosen for injection of tau in these experiments given the potent reduction in contrast agent infiltration observed in the above magnetic resonance experiments, and the relatively large and homogenous nature of this subcortical brain structure. CSF was then collected from the cisterna magna, 15, 30, or 60 min later to determine the extent of clearance into CSF compartments. Reduced levels of total and phosphorylated (pS_199_) tau were detected in CSF following TGN-020 treatment [e.g. 15min post-striatal infusion, TGN-020 treated animal CSF total and phosphorylated (pS_199_) tau content, 0.09 ng/ml (±0.02 ng/ml) and 0.02 ng/ml (±0.01 ng/ml) respectively, versus 1.01 ng/ml (±0.25 ng/ml) and 0.30 ng/ml (±0.08 ng/ml) in vehicle-treated animals, *P *<* *0.0001 in both comparisons] (Fig. 6F and G). Similar experiments were also performed in *Aqp4*^−/−^ mice, where vehicle or TGN-020 was given 15 min prior to intrastriatal infusion of tau containing brain homogenate and CSF extracted 30 min later. Similar to TGN-020 treated wild-types (*P *<* *0.001), *Aqp4*^−/−^ vehicle treated mice showed a reduction in CSF tau compared to vehicle-treated wild-types [*Aqp4*^−/−^ mice 0.07 ng/ml (±0.01 ng/ml) versus 0.63 ng/ml (±0.13 ng/ml) in wild-type mice, *P *<* *0.001] (Fig. 6H), further suggesting that glymphatic clearance of tau depends on AQP4. In addition, when *Aqp4*^−/−^ mice were treated with TGN-020, no further inhibition of tau clearance was observed [vehicle and TGN-020 treated *Aqp4*^−/−^ mice CSF total tau, 0.06 ng/ml (±0.01 ng/ml) and 0.10 ng/ml (±0.02 ng/ml), respectively] (Fig. 6H), indicative of the specific nature of this agent for AQP4 and the dependence of glymphatic function on this water channel.

Together these data suggest the specific nature of TGN-020 as an AQP4 inhibitor, and the imperative nature of AQP4 function for CSF-ISF exchange and clearance of tau from the mouse brain. This suggests that the observed perturbation in AQP4 polarization observed in the caudal cortex of rTg4510 mice may be a critical factor in the impairment of glymphatic inflow, and clearance of tau from this brain region in the rTg4510 mouse.

## Discussion

Here we demonstrate that in a tauopathy model, the cortical accumulation of tau is not solely allied to regional promoter activity, raising the question as to whether the glymphatic system may be involved in regulation of tau clearance in Alzheimer’s disease. As such, we investigated the healthy mouse brain to establish if there was a pattern of glymphatic clearance that could account for the regional nature of cortical tau deposition. We demonstrated that CSF-ISF exchange was lower in the rostral cortex, which was associated with accelerated tau deposition in Alzheimer’s disease mice. Quantifying glymphatic clearance in the rTg4510 mouse, we observed that CSF-ISF exchange and clearance of tau became impaired in the caudal cortex. In concert, we demonstrated that the level of AQP4 polarization to astrocytic endfeet was reduced in regions where glymphatic inflow and clearance of tau were impaired. Finally, we showed a marked reduction of CSF-ISF exchange and tau clearance with pharmacological inhibition of AQP4. Together these data suggest that glymphatic CSF-ISF exchange, and its role in clearance of tau from the brain, are impaired in this tau Alzheimer’s disease model, and that blocking AQP4 leads to decreased tau clearance, supporting pharmacological targeting of AQP4 for glymphatic modulation in tauopathic dementias such as Alzheimer’s disease.

Our initial finding of heterogenous tau deposition in the rTg4510 mouse cortex is counter-intuitive given the homogenous expression of the pathology-driving promoter, CaMK2a, in the mouse cortex. However, our subsequent findings of lower glymphatic inflow and clearance of tau in the rostral compared to caudal mouse cortex, suggest that CSF-ISF exchange may play a role in regional clearance, and therefore the deposition of tau in this animal model of tauopathy. The transport capacity of the glymphatic system has been shown to be driven by arterial pulsation (Iliff *et al.*, 2013*b*), and therefore regional differences in CSF-ISF exchange observed here in the cortex may be attributable to discrepancies in arterial architecture and input. Correspondingly, Xiong *et al.* (2017) showed that what we define as the rostral cortex receives ∼30% of the arterial input of the anterior cerebral artery and ∼40% of the middle cerebral artery. Whereas the caudal aspect of the cortex receives arterial input from both of these sources (∼10% and ∼30%, respectively) as well as the posterior cerebral and superior cerebellar arteries, and the anterior choroidal artery (Xiong *et al.*, 2017). Added to this, the hippocampus receives only ∼10% of the arterial input of the posterior cerebral and superior cerebellar arteries, and only ∼0.1% of the input from the anterior choroidal artery (Xiong *et al.*, 2017), in line with the low level of glymphatic inflow similarly observed in this brain region. In the current study we have not addressed whether such a low level of fluid exchange in the hippocampus also translates to a low extent of tau clearance, yet these data raise the suggestion as to the susceptibility of the hippocampus, like the rostral cortex, to deposition of tau protein. As can be seen in Fig. 1, rTg4510 animals exhibiting early tau pathology in the rostral cortex (e.g. ∼3.2 months, Fig. 1C), also exhibit tau deposition in the subiculum of the hippocampus (Fig. 1, arrows), which worsens with age to affect the entire structure (∼4.6 months, Fig. 1E). If the regional nature of CSF-ISF exchange observed here in the mouse brain translates across species to the human, this would correlate with the neuropathological picture of tau pathology in Alzheimer’s disease, as these brain regions present with tau pathology in the earliest stages of disease progression (Braak and Braak, 1995).

In the cohort of rTg4510 mice examined in our study, we observed subtle increases in CSF-ISF exchange and tau clearance. We hypothesize that these effects are due to neurodegeneration as a result of NFT pathology. For example, the rostral cortex and hippocampus, which are both markedly burdened by tau pathology (Fig. 1E), present with subtle increases in fluid exchange compared to wild-types. Conversely, in the cerebellum, which is void of tau pathology (Ramsden *et al.*, 2005), no differences were observed in contrast agent infiltration between wild-type and rTg4510 cohorts. Previously it has been observed that as glymphatic function declines with age (Kress *et al.*, 2014), so does extracellular volume (Syková *et al.*, 2005). We hypothesize therefore that increased extracellular volume as a result of tau-induced neurodegeneration would result in elevated magnetic resonance contrast agent inflow, as we observe here. It remains unclear, however, as to whether the subtle increase in glymphatic inflow would have any marked neurophysiological consequence in terms of elevated glymphatic clearance of toxic protein species in an already significantly degenerated scenario.

Consistent with data from others (Iliff *et al.*, 2014), our findings suggest that tau, in the extracellular space, is a substrate for clearance via the glymphatic pathway. Furthermore recent observations have suggested that tau, using the extracellular space as a conduit, is able to propagate from neuron-to-neuron via a ‘prion’-like mechanism (Chesser *et al.*, 2013; Ahmed *et al.*, 2014; Medina and Avila, 2014; Clavaguera *et al.*, 2015; Lewis and Dickson, 2016; Wu *et al.*, 2016; Goedert *et al.*, 2017). The functional relevance of appropriate glymphatic clearance of extracellular tau may therefore be of prime importance in neurodegenerative tauopathies, in which propagating pathology are thought to occur. An additional consideration to take is that once tau is cleared from the parenchyma into the CSF sink, the recirculating nature of CSF in the glymphatic clearance system suggests that tau-laden CSF may be re-internalized into the peri-arterial space and parenchyma (Nedergaard, 2013). In traumatic brain injury for example, a 40 000-fold increase of CSF tau is observed following injury (Zemlan *et al.*, 2002), resulting in typical paravascular predominance of tau-immunoreactive neurofibrillary tangles (Goldstein *et al.*, 2012). If CSF tau possesses the ability to ‘re-infect’ the brain, it raises the vital role of effective absorption methods of tau at the CSF–blood barrier and arachnoid villi, through the cervical lymphatic system, and the recently discovered meningeal lymphatics (Louveau *et al.*, 2015, 2017), in addition to appropriate function of the glymphatic clearance system.

Part of the aquaporin family of integral membrane proteins, AQP4 is the primary facilitator of transmembrane water transport in the brain, lowering the driving forces for fluid transport across plasma membranes (Nagelhus and Ottersen, 2013; Verkman *et al.*, 2017). Supportive of this notion that AQP4 supports and facilitates intraparenchymal fluid flow, AQP4 deletion potentiates increases in intracranial pressure (ICP) in a mouse model of hydrocephalus (Bloch *et al.*, 2006). AQP4 is expressed highly on the astrocytic endfeet, which face the perivascular space and is hence strategically positioned to allow rapid CSF and ISF exchange. Glymphatic flow of CSF into the brain’s interstitium and its clearance out of the parenchyma, is therefore thought to be facilitated by this localized expression and polarization of AQP4 to the endfeet of astrocytes surrounding blood vessels in the brain (Mestre *et al.*, 2018). Original data supporting this hypothesis came from the use of AQP4 null mice, in which glymphatic clearance was shown to be impaired by ∼70% compared to wild-type animals, resulting in a ∼55% reduction in amyloid-β clearance from the brain (Iliff *et al.*, 2012). Despite facing controversy (Jin *et al.*, 2016; Smith and Verkman, 2018) and concern relating to lack of reproducibility (Smith *et al.*, 2017), several independent groups have now similarly demonstrated that astrocytic AQP4 is essential for fast glymphatic transport, using various different approaches of *AQP4* gene deletion (Plog *et al.*, 2015; Achariyar *et al.*, 2016; Lundgaard *et al.*, 2016; Murlidharan *et al.*, 2016). In our current study, we have taken a different approach, and demonstrate that by pharmacologically inhibiting AQP4 function we observed a marked effect on glymphatic function; pharmacological inhibition resulted in a ∼85% reduction in MRI quantified CSF-ISF exchange, and a similar reduction in tau clearance from the brain. This effect is even more striking given previous evidence of cerebral blood flow (CBF) elevation (∼20%) in the mouse brain upon TGN-020 treatment (Igarashi *et al.*, 2013), and the opposing effect this would have upon glymphatic clearance given the intrinsic link between CBF, arterial pulsatility, and CSF-ISF exchange (Iliff *et al.*, 2013*b*; Bedussi *et al.*, 2016). Herein, our findings demonstrate the importance of AQP4 for appropriate glymphatic clearance from the brain parenchyma, alongside studies using genetic deletion approaches (Iliff *et al.*, 2012; Plog *et al.*, 2015; Achariyar *et al.*, 2016; Lundgaard *et al.*, 2016; Murlidharan *et al.*, 2016). Our data suggest a role for AQP4-mediated clearance of tau from the brain, which therefore implies its importance in neurodegenerative tauopathies such as Alzheimer’s disease (Xu *et al.*, 2015).

We observed here that AQP4 mRNA and protein expression levels are increased in the brains of rTg4510 mice, which is consistent with data from other degenerative Alzheimer’s disease scenarios (Yang *et al.*, 2011; Matarin *et al.*, 2015; Xu *et al.*, 2015). The Nedergaard laboratory suggest, however, that in addition to its appropriate expression in the brain, AQP4 must also be suitably polarized to astrocytic endfeet, in order to facilitate appropriate CSF-ISF exchange (Kress *et al.*, 2014). Reactive astrogliosis, though common place in degenerative Alzheimer’s disease phenotypes, is associated with increased AQP4 expression, and reduced AQP4 polarization (Iliff *et al.*, 2014; Kress *et al.*, 2014). Hence it is likely that reactive astrogliosis in the cortex of rTg4510 mice is responsible for the upregulations of AQP4 mRNA and protein levels observed. Yet given the phenotype of reduced polarization in such astrocytes, these upregulated expressions do not result in respective increases in AQP4 polarization, nor significantly elevated fluid exchange in the inflamed brain. Moreover, in the caudal cortex of rTg4510 mice, upregulation of AQP4 mRNA resulted in only subtle elevation in AQP4 protein, and significant reduction in both AQP4 polarization and CSF-ISF exchange. This questions the integrity of the membrane binding complex in astrocytes within this region, which facilitate AQP4 endfeet localization (Amiry-Moghaddam *et al.*, 2004; Camassa *et al.*, 2015). Studies from the Iliff laboratory have implicated α-syntrophin, an element of the AQP4 membrane binding complex, to be vital for glymphatic clearance (Mestre *et al.*, 2018), in that α-syntrophin knockout mice, despite expressing AQP4 at normal levels (Amiry-Moghaddam *et al.*, 2003), present with impaired glymphatic function. Further study of astrocytes in the caudal cortex of rTg4510 mice would therefore shed light on to the root cause of impaired glymphatic clearance in this model, and whether or not membrane complex elements such as α-syntrophin, or their PDZ binding domains, are affected (Camassa *et al.*, 2015) in degenerative tauopathy. To our knowledge, the data presented herein represent the first demonstration of perturbed AQP4 polarization in a tau model of Alzheimer’s disease. Taken together with similar findings in an amyloid-β model (Yang *et al.*, 2011), in aged mice (Kress *et al.*, 2014), and also in human Alzheimer’s disease (Zeppenfeld *et al.*, 2016), this research points to AQP4 as a novel therapeutic target for Alzheimer’s disease.

To assess glymphatic CSF-ISF exchange in the mouse brain using MRI, we adapted a previously published protocol for measurements in the rat brain (Iliff *et al.*, 2013*a*), by scaling down both the volume (80 μl in rat, 30 μl in mouse) and flow rate (1.6 μl/min in rat, 0.6 μl/min in mouse) at which intrathecal magnetic resonance contrast agent is administered. Our findings parallel patterns of glymphatic flow described in the brain using similar contrast infusion techniques in rodents (Iliff *et al.*, 2013*a*; Gaberel *et al.*, 2014). One technical challenge in the study of CSF-ISF exchange is the necessity to deliver contrast agents directly into the brain, leading to, for example, elevation of ICP brought about by the infusion itself. In the rat brain, intrathecal infusion has only been shown to increase ICP at ≥3 µl/min (Yang *et al.*, 2013; Bedussi *et al.*, 2016). Therefore, studies of the glymphatic system using intracisternal infusion rates of 1.6 µl/min in the rat brain are unlikely confounded by altered ICP (Iliff *et al.*, 2013*a*). In our studies of the mouse brain, we scaled down this infusion rate even further to 0.6 μl/min, and hence do not anticipate confounding of measurements. Furthermore, we apply two distinct methodologies to measure clearance: MRI of glymphatic inflow, and direct parenchymal injection, and observe good consistency between these different measures. The latter method of assessment of clearance, i.e. tau injection and quantification in extracted CSF, is itself prone to a relatively high degree of variability. This may stem from numerous factors, including the accuracy of microinjection, its location in relation to CSF extraction, the efficiency and purity (lack of blood contamination) of CSF extracts, and the accuracy and sensitivity of tau detection methods. Notwithstanding, we observe good alignment of data between these two complimentary methodological approaches, which strengthens the credibility of the findings presented here.

Here we provide the first description of changes in glymphatic CSF-ISF exchange, and clearance of tau from the brain of a mouse model of tauopathy, and suggest that the decline in function observed may be capable of exacerbating, or driving accumulation of tau in the brain, and by extension, neurodegeneration in Alzheimer’s disease. Our finding of impaired AQP4 polarization in affected regions of the tauopathy brain, and the effect of pharmacological AQP4 inhibition, suggest that this water channel may present as a novel target for modulation of this pathway for therapeutic effect in Alzheimer’s disease.

## Funding

This work was funded by research grants from Eli Lilly and Company, and the Engineering and Physical Sciences Research Council (EPSRC) UK (EP/N034864/1). I.H. is supported by Alzheimer’s Research UK (ARUK-RF2019A-003) and Parkinson’s UK (F-1902) fellowships. J.W. is supported by a Wellcome Trust/Royal Society Sir Henry Dale Fellowship (204624/Z/16/Z). M.F.L. receives funding from the EPSRC (EP/N034864/1); the King’s College London and UCL Comprehensive Cancer Imaging Centre CR-UK & EPSRC, in association with the MRC and DoH (England); UK Regenerative Medicine Platform Safety Hub (MRC: MR/K026739/1); Eli Lilly and Company.

## Competing interests

This work was part funded by a research grant from Eli Lilly and Company, of which Z.A., A.F., S.M., T.K.M. and M.J.O’N. are/were paid members of staff.

## Supplementary Material

awaa179_Supplementary_Data

## Abbreviations

ISF =: interstitial fluid
