# Supplementary material for: Impaired glymphatic function and clearance of tau in an Alzheimer’s disease model
Source: Brain. 2020 Jul 23;143(8):2576–93. doi: 10.1093/brain/awaa179 (PMC7447521; doi:10.1093/brain/awaa179)

Supplementary Figure 1. Spatial and Temporal Profile of Glymphatic Inflow in the Mouse Brain.

(**A**) Timeline of imaging experiments used to measure glymphatic function in the mouse brain, through cisterna magna infusion of Gd-DTPA, and serial acquisition of T1-weighted MR images for 3 hours. (**B**) Representative psuedocolour scaled sagittal (~0.5mm lateral of bregma) images of the mouse brain after cisterna magna infusion of Gd-DTPA, showing infiltration of contrast agent into the brain parenchyma. Scale bar equal to 1mm. (**C**) Schematic illustrating direction of contrast agent flow into the brain following cisterna magna infusion, shown temporally based on extracted best-fit $Time_{50}$ values (**D**) (shown graphically in **F**) derived from sigmoidal fitting of MRI T1 signal intensity vs. time data (**E**). Data shown as mean ± SEM between animals in **E**, and as best-fit value and associated 95% confidence interval of sigmoidal fitting of data in **F**. N=5.

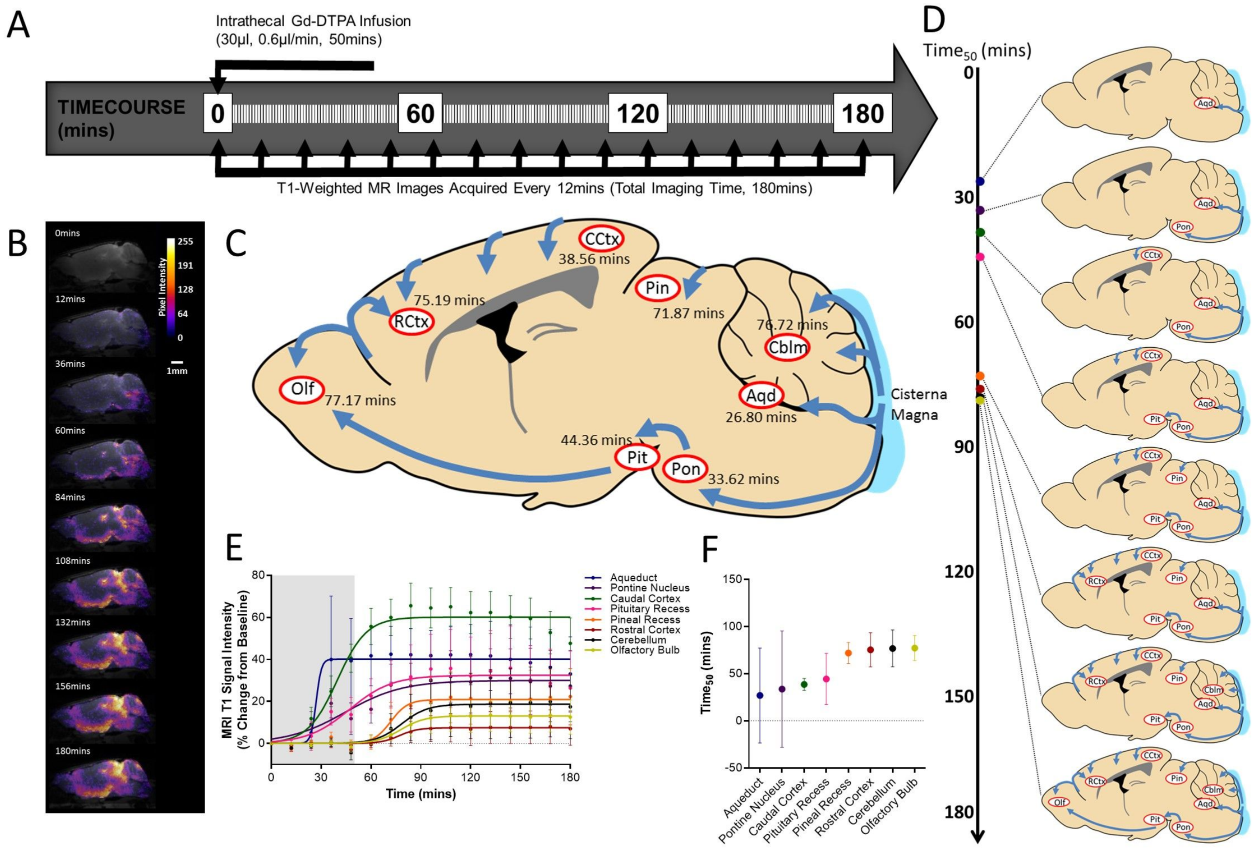

Supplementary Figure 2. Reactive Astrogliosis in the Brains of rTg4510 Mice.
(**A**) Representative example images of brain tissue from wildtype and rTg4510 mice immunohistochemically stained for GFAP (an astrocyte marker). Arrows indicate examples of immunopositive cells in each image. Immunoreactivity quantification shown in (**C**), which broadly reflects mRNA expression in each of the 4 regions examined (**B**). N=3-5 per group. Statistical significance denoted by asterisks: **=p<0.01, ***=p<0.001, ****=p<0.0001.

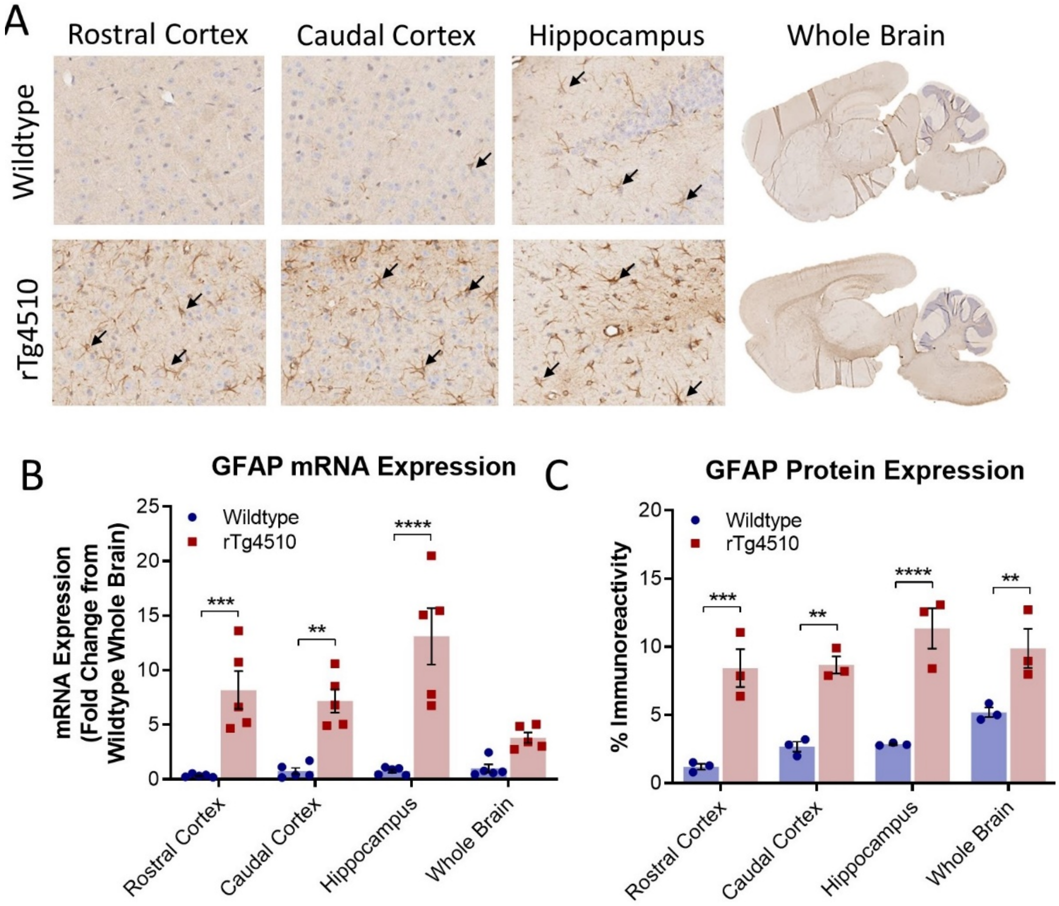

Supplement: awaa179_Supplementary_Data [file awaa179_supplementary_data.pdf]
